# Two Decades of Childhood Ependymoma Experience at a Tertiary Cancer Center in Turkey

**DOI:** 10.3390/children13060765

**Published:** 2026-05-30

**Authors:** Selma Cakmakci, Harun Demirci, Gonca Altinisik Inan, Servet Guresci, Suheyla Aytac Arslan, Arzu Yazal Erdem, Derya Ozyoruk, Ibrahim Qaddoumi, Inci Ergurhan Ilhan, Neriman Sari

**Affiliations:** 1 Department of Pediatric Hematology and Oncology, Ankara Bilkent City Hospital, Ankara 06800, Türkiye; arzu.yazalerdem@sbu.edu.tr (A.Y.E.); derya.ozyoruk@sbu.edu.tr (D.O.); ergurhani@gmail.com (I.E.I.); neriman.sari@sbu.edu.tr (N.S.); 2 Department of Neurosurgery, Ankara Bilkent City Hospital, Ankara 06800, Türkiye; harundemirci@aybu.edu.tr; 3 Department of Radiation Oncology, Ankara Bilkent City Hospital, Ankara 06800, Türkiye; goncaaltinisikinan@aybu.edu.tr (G.A.I.); suheylaaytacarslan@aybu.edu.tr (S.A.A.); 4 Department of Pathology, Ankara Bilkent City Hospital, Ankara 06800, Türkiye; servet.guresci1@saglik.gov.tr; 5 Department of Oncology, St. Jude Children’s Research Hospital, Memphis, TN 38105, USA; ibrahim.qaddoumi@stjude.org

**Keywords:** ependymoma, children, survival, pediatric brain tumor, gross total resection, LMIC

## Abstract

**Highlights:**

**What are the main findings?**

- In a single-center cohort of 32 pediatric ependymoma patients followed between 2006 and 2024, gross total resection (GTR) was the most significant prognostic factor, with 3-year EFS of 79.0% in GTR versus 38.9% in subtotal resection (STR) (*p* = 0.009), and 3-year OS of 100% versus 79.4% (*p* = 0.035).
- Overall survival and event-free survival rates were comparable to those reported in the international literature, with four deaths (12.5%) recorded during the follow-up period.

**What are the implications of the main findings?**

- Maximum safe surgical resection should remain the primary treatment goal in pediatric ependymoma, as GTR is strongly associated with improved survival outcomes.
- In resource-limited settings, multimodal therapy including chemotherapy and radiotherapy at relapse may offer a survival benefit and warrants further prospective investigation.

**Abstract:**

Purpose: We aim to review our experience in treating children with ependymoma, the third most common malignant central nervous system tumor in children, at Ankara Bilkent City Hospital. Methods: We reviewed medical records of children <18 years old at diagnosis with ependymoma followed up between 2006 and 2024. Clinical, pathological, radiological, treatment, and outcome data were evaluated. Results: Thirty-two patients (56% males) were included. Median age at diagnosis was 6.8 years (range: 0.6–17 years). Sixteen tumors (50%) were Grade 2 histology. Resection extent was gross total resection (GTR, *n* = 16), subtotal resection (STR, *n* = 15), or biopsy (*n* = 1). Radiotherapy was given to 10 patients; chemotherapy to 3; and both to 11. Eight patients underwent surgery only. In univariate analysis, resection extent significantly impacted both event-free survival (EFS) (3-year EFS 79.0% in GTR vs. 38.9% in STR, *p* = 0.009) and overall survival (3-year OS 100% in GTR vs. 79.4% in STR, *p* = 0.035). Four patients (12.5%) died. Six patients remained alive with active disease; three were lost to follow-up. Conclusions: The best outcomes occurred in patients who underwent GTR. The EFS/OS rates were comparable to those in the literature. Our findings suggest that chemotherapy and radiotherapy in relapsed ependymoma may prolong survival.

## 1. Introduction

Ependymomas are the third most common malignant brain tumors in children, comprising approximately 9% of all childhood central nervous system (CNS) tumors and can occur throughout the brain and spine. They are most frequent in the posterior fossa, followed by the supratentorial region, and less commonly found in the spinal area [1].

The standard treatment approach involves surgery with maximum safe resection, followed by focal radiotherapy (RT) to the tumor bed [2]. The prognosis of intracranial ependymomas largely depends on the extent of resection in cases of localized disease [3,4]. The role of chemotherapy remains unclear and controversial. Several systematic reviews have investigated the efficacy of chemotherapy in the management of newly diagnosed ependymoma; however, none have provided conclusive evidence of a survival benefit [5,6,7,8,9,10].

In low- and middle-income countries (LMICs), the management of ependymoma is substantially more challenging than in high-income settings. Specific barriers include delayed diagnosis due to limited neuroimaging availability, reduced neurosurgical expertise and limited access to intraoperative neuronavigation technologies, restricted or delayed access to focal radiotherapy, and the complete unavailability of proton beam therapy—an emerging modality offered at select high-income institutions. Although chemotherapy protocols are broadly similar across settings, the ability to deliver timely and complete treatment sequences is frequently compromised by resource constraints [6,11]. Whether the outcomes achievable at LMIC centers are comparable to those reported from high-income institutions remains an important and underexplored question.

In this study, we aim to present a comprehensive overview of our 20-year institutional experience with pediatric ependymoma in an LMIC setting, highlighting diagnostic and therapeutic challenges, treatment outcomes, and areas for improvement.

## 2. Patients and Methods

We retrospectively reviewed medical records of patients aged 0 to 18 years diagnosed with ependymoma and treated at the Pediatric Oncology Department of Ankara Bilkent City Hospital between October 2006 and December 2024. Data on demographic characteristics, presenting symptoms, tumor features, histopathological findings, surgical approaches, treatment modalities, treatment responses, and survival outcomes were reviewed and recorded. Molecular subgroup analysis for posterior fossa tumors using H3K27me3 immunohistochemistry became available only after 2020; therefore, molecular classification could be performed only in a subset of patients. Ethics committee approval was obtained from Ankara Bilkent City Hospital Ethics Committee (Approval date: 30 July 2025, No.: 1-25-1537), and the study was conducted in accordance with the principles of the Declaration of Helsinki.

Treatment outcomes were assessed in terms of event-free survival (EFS) and overall survival (OS). EFS was defined as the time from diagnosis to either the first event or the last follow-up, and OS as the time from diagnosis to either death or the last known follow-up. The Kaplan–Meier method was used to generate survival curves. Events evaluated included: recurrence, treatment-resistant disease, secondary malignancy, or death.

Univariate comparisons between different patient subgroups were performed using the log-rank test. Cox proportional hazards regression analysis was used to evaluate the influence of various factors on survival and to calculate hazard ratios (HRs). The Chi-square test was applied to compare the distribution of clinical variables at diagnosis.

## 3. Results

A total of 43 patients were treated for ependymoma during the study period. Eleven patients were excluded because of missing data; the remaining 32 patients (18 male, 14 female) were included in the analysis. The median age at diagnosis was 6.8 years (range: 0.6–17 years) (Table 1). The most common presenting symptoms were headache and vomiting 15/32 (47%), followed by motor weakness (19%) and back pain (16%). The median duration from symptom onset to diagnosis was 30 days (range: 7–180 days).

**Figure 1:**
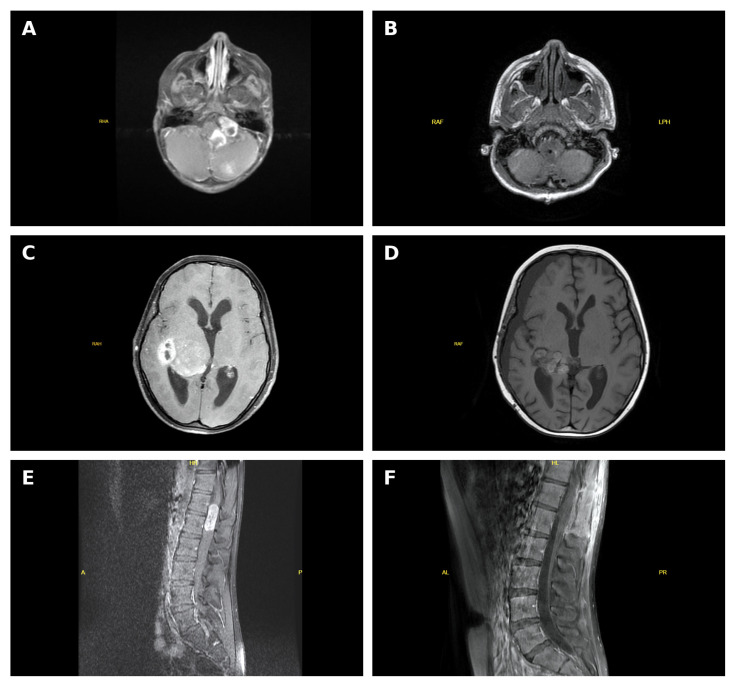
Representative magnetic resonance imaging findings of pediatric ependymoma according to tumor location. (**A**,**B**) Posterior fossa ependymoma. Preoperative contrast-enhanced MRI demonstrates a posterior fossa mass, and postoperative imaging shows the resection cavity. (**C**,**D**) Supratentorial ependymoma. Preoperative axial MRI demonstrates a supratentorial lesion, while postoperative MRI shows postsurgical changes after tumor resection. (**E**,**F**) Spinal ependymoma. Preoperative sagittal MRI demonstrates an intradural spinal lesion, and postoperative MRI shows postoperative changes following resection.

All patients underwent cranial magnetic resonance imaging (MRI) at diagnosis and follow-up MRI within 48 h after surgery to assess the extent of resection. Representative MRI examples illustrating posterior fossa, supratentorial, and spinal ependymomas, together with postoperative appearances, are presented in Figure 1.

Spinal MRI was performed in all cases as part of staging. Cerebrospinal fluid (CSF) analysis was conducted in 17 patients (53%).

Nineteen patients (60%) had tumors in the posterior fossa; 7 (21%) had supratentorial tumors, and 6 (19%) had spinal tumors. The histological subtypes were anaplastic in 14 patients (44%), classic in 15 patients (47%), and myxopapillary in 3 patients (9%). Sixteen tumors (50%) were classified as WHO grade 2; 16 tumors (50%) were grade 3. Among the 19 patients with posterior fossa ependymoma, molecular subgroup analysis using H3K27me3 immunohistochemistry was available in 7 cases (37%), as this technique became routine practice at our institution from 2022 onward. Five tumors (71%) were classified as posterior fossa group A (PFA), characterized by loss of H3K27me3 expression, and two (29%) as posterior fossa group B (PFB), retaining H3K27me3 expression. Clinical characteristics and outcomes of molecularly classified patients are summarized in Table 2. Among PFA patients, four of five (80%) had grade 3 histology; one patient achieved complete remission, while four were alive with active disease at last follow-up. Both PFB patients had grade 2 histology and were disease-free at last follow-up. Molecular subgroup data did not prospectively influence treatment decisions; chemotherapy and radiotherapy planning was guided by histological grade, extent of resection, and patient age throughout the study period.

Tumor size data were available for 28 of 32 patients (87.5%); the remaining 4 patients lacked archival imaging from the early years of the study. Among patients with available data, the median tumor diameter was 41 mm (range: 20–120 mm). No statistically significant difference in 3-year EFS was observed between tumors ≤ 41 mm and those > 41 mm (54.4% vs. 63.5%; log-rank *p* = 0.955), though this analysis was limited by missing data in four patients.

Spinal metastasis was observed in only four patients (12%). Among the 17 patients who underwent CSF analysis, malignant cytology was detected in 1 case (6%). All patients underwent surgical intervention. Gross total resection (GTR) was achieved in 16 patients (50%); subtotal resection (STR) was performed in 15 patients (47%), and one patient underwent tumor biopsy only (3%). A second-look surgery was performed in five patients, and GTR was achieved during the second procedure in three of these cases.

Radiotherapy was administered to 21 patients (66%), including 16 with focal RT, 2 with craniospinal irradiation (CSI), and 2 with cranial irradiation. The median radiotherapy dose was 54.9 Gy (range: 36–60 Gy). The median interval between surgery and the initiation of radiotherapy was 40 days (range: 14–90 days). Nine patients received RT at relapse.

Chemotherapy was administered as adjuvant treatment in 14 patients (44% of the cohort), 8 of whom were younger than 3 years of age. Alternating cycles of vincristine/cyclophosphamide and carboplatin/etoposide were most commonly used (*n* = 8). The median number of chemotherapy cycles was eight (range: 3–12). Eight patients received chemotherapy at relapse.

### Outcomes

At a median follow-up of 48.5 months (range: 5–150 months), events were observed in 16 patients (50%), including 4 progressions and 12 relapses. Six patients experienced metastatic relapse, and six experienced local relapse. The median time to relapse was 22 months (range: 3–59 months).

Among the 16 patients who experienced relapse/progression, 11 underwent relapse surgery. Of these 11 patients, 9 also received re-irradiation (RT2). The median time from the initial radiotherapy to RT2 was 25 months (range: 8–43 months). The median OS of the nine patients who received RT2 was 58 months (range: 13–91 months).

**Figure 2:**
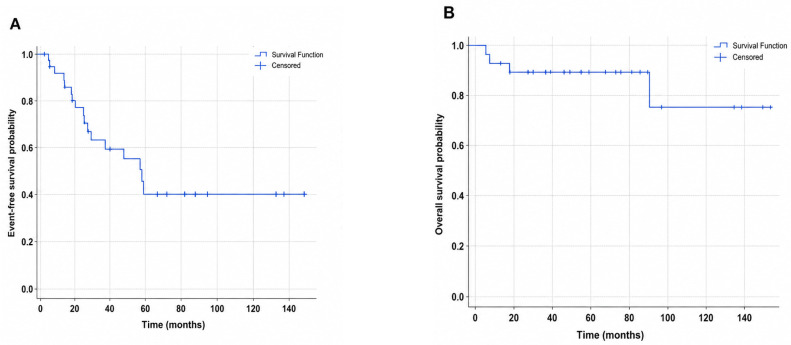
Kaplan–Meier survival curves for the overall cohort (*n* = 32). (**A**) Event-free survival. At 3 years, the EFS rate was 60.4%. (**B**) Overall survival. At 3 years and 5 years, the OS rate was 90.4%. Tick marks indicate censored observations.

The 3-year EFS and OS rates for the overall cohort were 60.4% (Figure 2A) and 90.4% (Figure 2B), respectively. The respective 3-year EFS and OS rates were both 100% in patients with spinal tumors, 57.1% and 85.7% in those with supratentorial tumors, and 51.3% and 89.2% in those with posterior fossa ependymomas (*p* = 0.417).

In univariate analysis, the only factor significantly affecting EFS was the extent of resection. The 3-year EFS rate was 79.0% in patients who underwent GTR and 38.9% in those who underwent STR (log-rank *p* = 0.009). The corresponding 3-year OS rates were 100% in the GTR group and 79.4% in the STR group (log-rank *p* = 0.035). This effect on EFS was confirmed in multivariable analysis (STR: RR = 2.4, 95% CI: 1.3–2.6, *p* = 0.005). Overall, four deaths (12.5%) occurred, all in patients who had undergone STR. Six of the surviving patients (21.4%) were alive with active disease, and three (10.7%) were lost to follow-up at last contact (Table 3).

Location-based treatment characteristics and outcome data are provided in Table 4.

Post-surgical complications were documented in 15 of 32 patients (46.9%). Hydrocephalus requiring ventriculoperitoneal (VP) shunt placement occurred in four patients (12.5%), all of whom had posterior fossa ependymoma. New neurological deficits including hemiparesis, hemiplegia, or reduced level of consciousness were observed in four patients (12.5%) in the immediate postoperative period. Wound dehiscence occurred in four patients (12.5%), and cerebrospinal fluid leak requiring lumbar drainage in two patients (6.3%). Central nervous system infection (meningitis or wound abscess) was documented in two patients (6.3%). A Grade 1 facial nerve deficit was noted in one patient (3.1%). Intensive care unit admission was required in three patients (9.4%), all in the context of advanced or refractory disease. Permanent complications were recorded in two patients (6.3%): diabetes insipidus in one patient and home ventilator dependency in another.

## 4. Discussion

In this study, we highlight the numerous challenges encountered while managing ependymoma in LMICs and draw attention to real-world data that reflect the complexities of treatment delivery in resource-constrained settings. The most significant finding was that, consistent with the existing literature, patients with ependymoma who underwent GTR had the most favorable survival outcomes [12,13,14]. In our cohort, 47% of patients underwent STR during their initial surgery, and 73% of these incompletely resected tumors were in the posterior fossa. The median age at diagnosis was 9 years for patients who underwent GTR and 2.8 years for those who underwent STR. By comparison, gross total resection rates reported from high-income country series generally range from 50% to 75%, with the highest rates achieved at specialized neurosurgical centers equipped with intraoperative neuronavigation and neurophysiological monitoring [14,15]. The relatively high STR rate in our cohort likely reflects constraints inherent to the LMIC neurosurgical environment.

In the first prospective AIEOP study, the 5-year PFS and OS rates in 63 patients with pediatric ependymoma were reported as 56% and 75%, respectively [16,17]. Merchant et al. reported 7-year EFS and OS rates of 69% and 81% in a prospective series [14]. In the present cohort, the 3-year EFS and OS for intracranial ependymoma were 52.8% and 88.3%, respectively—findings broadly consistent with the published literature. Notably, six patients are currently living with active disease and three were lost to follow-up, all of whom remain at risk of eventual progression, and a longer observation period may refine long-term survival estimates.

A slight male predominance was noted in our cohort, aligning with previously reported epidemiological trends [18,19,20,21,22]. Patients with posterior fossa tumors had a notably lower mean age (4.2 years) than those with supratentorial (11.1 years) or spinal (11.9 years) ependymomas.

The median duration from symptom onset to diagnosis in our cohort was 30 days. For spinal tumors, the median duration was longer (50 days), consistent with the more insidious presentation of these lesions. Diagnostic delays may contribute to long-term complications such as visual impairment, endocrine dysfunction, and neurocognitive deficits [23,24].

In the ACNS0831 trial, outcomes were evaluated in patients with intracranial ependymoma who underwent gross-total or near-total resection, followed by postoperative focal RT alone or RT plus four cycles of maintenance chemotherapy. The as-treated analysis showed a significantly higher 3-year EFS in patients who received chemotherapy (80%) than in those who received RT alone (71%) [25]. In our cohort, although the number of patients receiving adjuvant chemotherapy was limited, no clear survival advantage was observed.

It has been reported that surgery alone is not sufficient for favorable outcomes in recurrent ependymomas [13,26,27,28], and several studies have shown that re-irradiation following surgery can provide a survival benefit. In our study, among the 16 patients who experienced relapse, only 9 had undergone re-irradiation. Although the number was small, we conclude that re-irradiation at relapse provided a survival benefit in our patient group.

In recent years, molecular classification has significantly improved the understanding of pediatric ependymoma biology and prognosis. The distinction between PFA and PFB tumors based on H3K27me3 immunohistochemistry carries important prognostic implications: PFA tumors predominantly affect young children and are associated with a substantially inferior prognosis, whereas PFB tumors tend to occur in older patients and carry a more favorable outcome [29]. In the present cohort, five of seven molecularly characterized posterior fossa tumors (71%) were classified as PFA consistent with the known predominance of this subgroup in pediatric series. The clinical course observed aligned with published prognostic data: four of five PFA patients (80%) were alive with active disease at last follow-up, whereas both PFB patients were in complete remission. These findings, while derived from a small molecularly characterized subset, support the adverse prognosis associated with PFA in the LMIC setting. Future protocols should incorporate prospective molecular stratification to enable subgroup-adapted treatment planning.

Importantly, despite the resource constraints inherent to our LMIC setting, including the absence of proton beam therapy, limited intraoperative navigation infrastructure, and historically restricted access to molecular profiling, the survival rates observed in the present cohort were broadly comparable to those reported from high-income-country institutions [14,16,17]. This finding underscores that adherence to core principles, maximal safe resection and timely focal radiotherapy can yield outcomes consistent with the international literature even in resource-limited environments.

This study has several important limitations. First, it was conducted retrospectively, which may have introduced biases related to data accuracy and completeness. Second, the number of patients included was relatively small, limiting statistical power. Third, molecular profiling was available only for a limited subset of patients diagnosed after 2022, precluding subgroup-stratified analyses for the majority of the cohort. Fourth, tumor size data were unavailable for 4 of 32 patients (12.5%) due to the inaccessibility of archival imaging from the early years of the study, which limited the evaluation of tumor diameter as a prognostic factor. Fifth, post-surgical complication data were available for 20 of 32 patients; the remaining 12 patients had no documented complications, which may reflect incomplete retrospective capture rather than true absence of adverse events. Sixth, the study spans an 18-year period (2006–2024), during which treatment approaches evolved substantially: in the earlier years, adjuvant chemotherapy was administered more liberally, including in patients who had undergone GTR with grade 3 histology, whereas in recent years it was omitted after GTR followed by focal radiotherapy. The age threshold for radiotherapy also shifted: while irradiation was historically avoided in children under 3 years of age, current institutional practice extends radiotherapy to children older than 12 months. This temporal heterogeneity in treatment strategies limits direct comparisons across the cohort. Seventh, although the follow-up period was extended prior to resubmission, the current median follow-up of 48.5 months remains relatively limited for a disease in which late relapses are well-documented, and the 3-year survival estimates reported may be revised with continued observation. Despite these limitations, our findings offer insight into real-world clinical practice in an LMIC setting and highlight the potential benefit of re-irradiation at relapse in select patients.

## 5. Conclusions

This study highlights the multifaceted challenges of managing pediatric ependymoma in resource-limited settings and underscores the prognostic importance of GTR. Our findings align with the existing literature in demonstrating that the extent of resection remains the most significant predictor of survival. Although re-irradiation at relapse appeared to offer a survival advantage in select patients, the benefit of chemotherapy remains uncertain. Continued efforts to improve surgical outcomes, ensure timely radiotherapy, and develop molecularly informed treatment approaches are essential to optimizing care for children with ependymoma, particularly in low- and middle-income countries.

## Figures and Tables

**Table 1:** Patient Demographics, Clinical Characteristics, and Treatment Details.

Patient ID	Sex	Age (y)	Location	Histology	Grade	Size (mm)	Metastasis	Surgery	2nd-Look	RT	RT Dose (Gy)	CT	CT Cycles
1	M	13.0	ST		2		Absent	GTR		Yes	54.0	Yes	8
2	M	10.0	ST	Classic	2	46.0	Absent	GTR		No		No	
3	F	15.0	ST	Anaplastic	3	70.0	Absent	GTR		Yes	59.4	No	
4	M	12.0	ST	Anaplastic	3	42.0	Absent	STR	Yes	Yes	60.0	Yes	7
5	F	11.0	ST	Anaplastic	3	53.0	Absent	STR		Yes	59.4	No	
6	M	8.0	ST	Anaplastic	3	65.0	Absent	STR		Yes	55.8	Yes	6
7	M	9.0	ST	Anaplastic	3	28.0	Absent	GTR		Yes	59.4	No	
8	M	1.6	PF	Anaplastic	3		Absent	GTR		Yes		Yes	
9	F	2.5	PF	Classic	2	55.0	Absent	GTR		No		No	
10	F	6.5	PF	Classic	2	38.0	Absent	GTR	Yes	No		No	
11	F	2.2	PF	Classic	2	40.0	Absent	GTR		Yes	45.0	No	
12	F	1.4	PF		2	33.0	Absent	STR	Yes	Yes	54.0	No	
13	F	7.0	PF	Classic	3	45.0	Absent	GTR		Yes	55.8	Yes	11
14	F	2.8	PF	Classic	3	56.0	Present	STR		Yes	54.0	Yes	6
15	F	2.5	PF	Classic	2	35.0	Absent	STR	Yes	Yes	50.4	No	
16	M	0.6	PF	Anaplastic	3	30.0	Present	STR		No		Yes	
17	F	2.5	PF	Anaplastic	3		Absent	STR		No		Yes	12
18	F	1.9	PF	Classic	2	40.0	Present	STR		Yes	57.6	Yes	7
19	M	13.0	PF	Classic	2	43.0	Absent	GTR		Yes	54.0	No	
20	M	13.0	PF		2		Absent	GTR		Yes	54.0	No	
21	M	4.0	PF	Anaplastic	2	40.0	Absent	STR		No		No	
22	F	1.0	PF	Anaplastic	3	47.0	Absent	STR		No		Yes	12
23	M	4.0	PF	Anaplastic	3	40.0	Absent	STR		Yes	54.0	Yes	3
24	M	2.8	PF		3	35.0	Absent	GTR	Yes	Yes	59.4	Yes	8
25	M	9.0	PF	Anaplastic	3	55.0	Absent	STR		Yes	60.0	Yes	10
26	M	2.7	PF	Anaplastic	3	50.0	Absent	STR		Yes	59.4	Yes	8
27	M	2.0	Spinal	Classic	2	20.0	Absent	GTR		No		No	
28	M	9.0	Spinal	Myxopapillary	2	40.0	Absent	GTR		No		No	
29	M	13.0	Spinal	Myxopapillary	2	56.0	Absent	GTR		No		No	
30	M	16.5	Spinal	Myxopapillary	2	38.0	Absent	GTR		No		No	
31	F	14.0	Spinal	Classic	2	24.0	Present	Biopsy		No	36.0	No	
32	F	17.0	Spinal	Classic	2	120.0	Absent	STR		No	50.4	No	8

Abbreviations: M, male; F, female; PF, posterior fossa; ST, supratentorial; GTR, gross total resection; STR, subtotal resection; RT, radiotherapy; CT, chemotherapy. Grade refers to WHO classification. Empty cells indicate missing or non-applicable data.

**Table 2:** Clinical characteristics and outcomes of molecularly classified posterior fossa ependymoma patients.

Patient ID	Age (y)	Grade	Surgery	Radiotherapy	Event	Current Status
PFA						
14	2.8	3	STR	Yes (54 Gy)	No	NED
18	1.9	2	STR	Yes (57.6 Gy)	Yes	Alive with disease
23	4.0	3	STR	Yes (54 Gy)	Yes	Alive with disease
24	2.8	3	GTR (2nd-look)	Yes (59.4 Gy)	Yes	Alive with disease
26	2.7	3	STR	Yes (59.4 Gy)	Yes	Alive with disease
PFB						
10	6.5	2	STR (2nd-look)	No *	No	NED
19	13.0	2	GTR	Yes (54 Gy)	No	NED

* “Radiotherapy was planned but not administered due to prolonged follow-up interruption; the patient was disease-free upon return and RT was withheld.” Abbreviations: PFA, posterior fossa group A; PFB, posterior fossa group B; GTR, gross total resection; STR, subtotal resection; NED, no evidence of disease.

**Table 3:** Patient Outcomes and Follow-Up Data.

Patient ID	Follow-Up (Months)	Event	Time to Event (Months)	Treatment at Relapse	RT1 to RT2 (Months)	Current Status	Survival (Months)
1	89	Absent	90	Absent		No evidence of disease	90.1
2	41	Absent	17	Absent		No evidence of disease	17.5
3	55	Absent	41	Absent		No evidence of disease	47.0
4	39	Absent	39	Absent		No evidence of disease	39.0
5	7	Progression	3	CT		Death	7.5
6	138	Recurrence	19	Surgery + CT		No evidence of disease	140.3
7	58	Recurrence	27	Surgery + RT	26	No evidence of disease	40.1
8	83	Absent	65	Absent		No evidence of disease	65.1
9	135	Absent	137	Absent		No evidence of disease	137.2
10	18	Absent	3	Absent		No evidence of disease	3.0
11	150	Absent	152	Absent		No evidence of disease	152.4
12	67	Absent	47	Absent		No evidence of disease	47.4
13	97	Absent	97	Absent		No evidence of disease	97.6
14	42	Absent	25	Absent		No evidence of disease	25.7
15	40	Absent	30	Absent		No evidence of disease	30.4
16	5	Progression	4	Absent		Death	5.4
17	18	Recurrence	16	Absent		Death	18.0
18	29	Progression	12	Absent		Alive with disease	13.2
19	28	Recurrence	25	Absent		Alive with disease	25.0
20	76	Recurrence	47	CT + RT	43	Lost to follow-up	76.7
21	13	Recurrence	7	Surgery + CT + RT		Lost to follow-up	13.3
22	90	Recurrence	58	Surgery + CT + RT		Death	90.0
23	46	Progression	13	Surgery + CT + RT	8	Alive with disease	32.0
24	36	Recurrence	17	Surgery + RT	18	Alive with disease	21.1
25	91	Recurrence	24	Surgery + CT + RT	24	Alive with disease	75.4
26	49	Recurrence	29	Surgery + CT + RT	28	Alive with disease	33.9
27	14	Absent	2	Absent		No evidence of disease	1.2
28	72	Absent	72	Absent		No evidence of disease	72.1
29	58	Recurrence	37	Surgery + RT		No evidence of disease	45.0
30	48	Absent	48	Absent		Lost to follow-up	48.0
31	27	Absent	12	Absent		No evidence of disease	12.0
32	26	Absent	9	Absent		No evidence of disease	9.0

Abbreviations: RT1, initial radiotherapy; RT2, re-irradiation; CT, chemotherapy; RT, radiotherapy.

**Table 4:** Treatment characteristics and outcomes stratified by tumor location.

	Posterior Fossa (*n* = 19)	Supratentorial (*n* = 7)	Spinal (*n* = 6)
GTR	8 (42%)	4 (57%)	4 (67%)
STR/Biopsy	11 (58%)	3 (43%)	2 (33%)
Radiotherapy	13 (68%)	6 (86%)	2 (33%)
Chemotherapy	11 (58%)	3 (43%)	0 (0%)
Spinal metastasis	3 (16%)	0 (0%)	1 (17%)
Events (relapse/progression)	11	4	1
Deaths	3	1	0
3-year EFS	51.3%	57.1%	100%
3-year OS	89.2%	85.7%	100%
Median follow-up, months (range)	46 (5–150)	58 (7–138)	37.5 (14–73)

## Data Availability

Source data are available from the corresponding author upon reasonable request due to ethical restrictions.
